# Examination of SARS-CoV-2 serological test results from multiple commercial and laboratory platforms with an in-house serum panel

**DOI:** 10.1099/acmi.0.000463.v4

**Published:** 2024-02-29

**Authors:** Sandra N. Lester, Megan Stumpf, Brandi D. Freeman, Lisa Mills, Jarad Schiffer, Vera Semenova, Tao Jia, Rita Desai, Peter Browning, Bailey Alston, Muyiwa Ategbole, Shanna Bolcen, Alexander Chen, Ebenezer David, Panagiotis Manitis, Heather Tatum, Yunlong Qin, Briana Zellner, Jan Drobeniuc, Alexandra Tejada-Strop, Payel Chatterjee, Punya Shrivastava-Ranjan, M. Harley Jenks, Laura K. McMullan, Mike Flint, Christina F. Spiropoulou, Glenn P. Niemeyer, Bonnie J. Werner, Christopher J. Bean, Jeffrey A. Johnson, Alex R. Hoffmaster, Panayampalli S. Satheshkumar, Amy J. Schuh, S. Michele Owen, Natalie J. Thornburg

**Affiliations:** ^1^​ Respiratory Viruses Immunology Team, CDC, Atlanta, Georgia, USA; ^2^​ Eagle Global Scientific, LLC, Atlanta, Georgia, USA; ^3^​ Microbial Pathogenesis & Immune Response Team, CDC, Atlanta, Georgia, USA; ^4^​ Division of Viral Hepatitis, National Center for HIV Viral Hepatitis STD and TB Prevention, Atlanta, Georgia, USA; ^5^​ Viral Special Pathogens Branch, CDC, Atlanta, Georgia, USA; ^6^​ Division of Blood Disorders, National Center on Birth Defects and Developmental Disabilities, CDC, Atlanta, Georgia, USA; ^7^​ National Center for HIV Viral Hepatitis STD and TB Prevention, Atlanta, Georgia, USA; ^8^​ Bacterial Special Pathogens Branch, DHCPP, CDC, Atlanta, Georgia, USA; ^9^​ Poxvirus and Rabies Branch, CDC, Atlanta, Georgia, USA

**Keywords:** antibodies, COVID-19, immunoassays, neutralization assay, SARS-CoV-2, serological tests

## Abstract

Severe acute respiratory syndrome (SARS) coronavirus 2 (SARS-CoV-2) is a novel human coronavirus that was identified in 2019. SARS-CoV-2 infection results in an acute, severe respiratory disease called coronavirus disease 2019 (COVID-19). The emergence and rapid spread of SARS-CoV-2 has led to a global public health crisis, which continues to affect populations across the globe. Real time reverse transcription polymerase chain reaction (rRT-PCR) is the reference standard test for COVID-19 diagnosis. Serological tests are valuable tools for serosurveillance programs and establishing correlates of protection from disease. This study evaluated the performance of one in-house enzyme linked immunosorbent assay (ELISA) utilizing the pre-fusion stabilized ectodomain of SARS-CoV-2 spike (S), two commercially available chemiluminescence assays Ortho VITROS Immunodiagnostic Products Anti-SARS-CoV-2 Total Reagent Pack and Abbott SARS-CoV-2 IgG assay and one commercially available Surrogate Virus Neutralization Test (sVNT), GenScript USA Inc., cPass SARS-CoV-2 Neutralization Antibody Detection Kit for the detection of SARS-CoV-2 specific antibodies. Using a panel of rRT-PCR confirmed COVID-19 patients’ sera and a negative control group as a reference standard, all three immunoassays demonstrated high comparable positivity rates and low discordant rates. All three immunoassays were highly sensitive with estimated sensitivities ranging from 95.4–96.6 %. ROC curve analysis indicated that all three immunoassays had high diagnostic accuracies with area under the curve (AUC) values ranging from 0.9698 to 0.9807. High positive correlation was demonstrated among the conventional microneutralization test (MNT) titers and the sVNT inhibition percent values. Our study indicates that independent evaluations are necessary to optimize the overall utility and the interpretation of the results of serological tests. Overall, we demonstrate that all serological tests evaluated in this study are suitable for the detection of SARS-CoV-2 antibodies.

## Data Summary

No new external data, tools, software, or code has been generated in this research. Raw experimental data is publicly available at https://data.cdc.gov/Laboratory-Surveillance/Examination-of-SARS-CoV-2-serological-test-results/hhvg-83jq [32].

## Introduction

Severe acute respiratory syndrome (SARS) coronavirus 2 (SARS-CoV-2), is a novel highly pathogenic human coronavirus that has emerged causing an ongoing global pandemic. SARS-CoV-2 is the causative agent of coronavirus disease 2019 (COVID-19), an acute respiratory disease that is associated with a wide range of respiratory illnesses and death [1–4]. Like other previously identified highly pathogenic coronaviruses, SARS-CoV-2 belongs to the *Betacoronavirus* genus [3, 5, 6]. Its emergence in the human population was first recognized in a cluster of patients with pneumonia in Wuhan, China in December 2019 [1–5]. Due to rapid spread, throughout the pandemic, infections of SARS-CoV-2 are detected worldwide. As of 26 July 2023, the World Health Organization (WHO) has reported 768 560 727 confirmed cases of SARS-CoV-2 infection globally, resulting in 6 952 522 deaths (https://covid19.who.int/) [7]. This global public health crisis has led to a continuous worldwide effort to rapidly identify infected populations and define antibody response profiles to SARS-CoV-2.

Clinical diagnosis of SARS-CoV-2 infection has primarily relied on the detection of viral RNA by real time reverse transcription polymerase chain reaction (rRT-PCR) of nasopharyngeal swabs or other clinical specimens from patients [8–10]. However, challenges remain with rRT-PCR testing that may yield false-negative results [10]. Serological tests that detect SARS-CoV-2-specific antibodies in serum or plasma specimens can assess past or recent infections from individuals [10, 11]. Serological tests can detect SARS-CoV-2-specific responses one to four weeks after infection or vaccination and are valuable tools for seroprevalence studies, assessing vaccine efficacies, and understanding correlates of immunity and protection.

The spike (S) protein and nucleocapsid (N) protein of coronaviruses (CoVs) elicit robust antibody responses in infected individuals [12–15]. The S glycoprotein of SARS-CoV-2 plays a pivotal role in SARS-CoV-2 infection by facilitating viral entry into host cells. The S glycoprotein is also the primary target of neutralizing antibodies, therapeutic drugs, and vaccines [12, 13, 16, 17]. Through the emergency use authorization (EUA) process, the Food and Drug Administration (FDA) authorized many serological tests primarily based on enzyme-linked immunosorbent assays (ELISA) and chemiluminescent immunoassays (CLIA) that detect the presence of antibodies against the SARS-CoV-2 S protein, its domains, or the N protein [18]. The antibody class targets of SARS-CoV-2 serology tests are IgM, IgG, IgA, and total antibody.

The specificity and sensitivity of serological tests determines their usefulness and interpretation of results. While the performance characteristics of some tests are available, independent comparative assessments are not widely available for all tests with an EUA from FDA. Furthermore, the correlation between qualitative assessment versus antibody quantification and binding antibody versus neutralizing abilities are not well known. Utilizing serological tests that will help accurately reflect estimates of SARS-CoV-2 prevalence and immunity in different populations is pivotal to the global public health response to the ongoing COVID-19 pandemic. The objective of this study was to evaluate the performance characteristics of multiple serology platforms and assess the correlation between each assay’s qualitative and quantitative values.

## Methods

### Cells

Vero cells (ATCC CCL-81) were cultured and maintained in Fluorobrite DMEM (Thermo Fisher Scientific) supplemented with 10 % (v/v) heat-inactivated fetal calf serum (HI-FCS) at 37 °C under a 5 % CO_2_ atmosphere. Cell lines were obtained from ATCC and authenticated by Cytochrome C oxidase, subunit I (CO1) analysis (Vero | ATCC). Mycoplasma testing was performed by Bionique Testing Lab using qPCR (Bionique Testing Laboratories | Mycoplasma Testing Services). Cell lines tested negative for Mycoplasma contamination.

### Patient specimens

Commercial serum specimens (BioIVT, StemExpress, and iSpecimen) sourced in 2020, were collected from 105 adult patients recruited based on their known SARS-COV-2 exposure status. Patients were laboratory confirmed negative or positive for SARS-CoV-2 by rRT-PCR. Of the 105 adult patients tested, 87 were rRT-PCR positive for SARS-CoV-2 and diagnosed with COVID-19. Serum specimens from these 87 rRT-PCR positive patients were used as the positive reference standard.

A total of 99 specimens collected before the COVID-19 pandemic, between October 2018–September 2019, and 18 rRT-PCR confirmed negative specimens served as the negative reference standard. Receiving laboratory randomized commercial specimens in the order they were received and generated identification numbers. Testing laboratories were blinded to the identity of specimens beyond the randomly assigned identification number.

### Immunoassay

#### ELISA Spike ectodomain in-house CDC ELISA

As previously described, the pre-fusion stabilized ectodomain of SARS-CoV-2 S ELISA was used to analyze antibody responses [19]. Briefly, the top half of Immulon 2 HB 96 well-plates (Fisher Scientific Cat. No 14-245-79) were coated with diluted purified spike protein, and the bottom half with PBS overnight at 4 °C in a humidified chamber. The next day, plates were washed using a BioTek plate 405 washer and blocked with 2.5X Stabilcoat blocking buffer. After blocking, plates were washed and 100 µl of serum diluent (PBS-T / 5 % skim milk) was added to all wells. A volume of 33.3 µl of serum diluted to 1 : 25 was added to rows A and E, and four-fold serial dilutions were performed. Horseradish peroxidase (HRP) conjugated goat anti-human antibodies (Seracare KPL Cat.No. 074–1007) diluted at 1 : 2000 in serum diluent were added to washed plates and incubated at 37 °C for 1 h. Plates were washed with PBS-T, 100 µl prepared substrate was added to each well, and were incubated for 30 min at 37 °C. After incubation, 100 µl prepared stop solution was added to each well, and plates were read at 405 and 490 nm using a PerkinElmer Victor XV plate reader. Final background corrected ODs were calculated as 490–405, antigen coated – PBS coated well for each dilution of each specimen. Specimens with OD values≥0.4 at a serum dilution of 1 : 100 are considered seropositive/reactive. Results are interpreted using a signal to threshold (S/T) value, an S/T value ≥1 is considered seropositive/reactive.

#### Ortho VITROS immunodiagnostic products Anti-SARS-CoV-2 total reagent pack assay

The Ortho VITROS Immunodiagnostic Products Anti-SARS-CoV-2 Total Reagent Pack assay was performed according to the manufacturer’s instructions (Ortho-Clinical Diagnostics Inc, Rochester, NY.) This is a chemiluminescent immunoassay test that qualitatively detects total antibody (including IgG, IgA and IgM) to SARS-CoV-2 S1 domain of S protein in human serum or plasma. The test received Emergency Use Authorization (EUA) from the Food and Drug Administration (FDA) and was subject to verification at the CDC for Clinical Laboratory Improvement Amendments (CLIA) regulated diagnostic testing. Results are interpreted as reactive for anti-SARS-CoV-2 total Ig if an index (S/C) is ≥1.0 and as non-reactive if <1.0.

#### Abbott SARS-CoV-2 IgG assay

The Abbott SARS-CoV-2 IgG assay was performed according to the manufacturer’s instructions (Abbott Laboratories Inc, Abbott Park, IL). This is a chemiluminescent microparticle immunoassay (CMIA) test that qualitatively detects IgG specific responses to the N protein in human serum or plasma. The presence or absence of IgG antibodies to SARS-CoV-2 in the sample is determined by comparing the chemiluminescent relative light units (RLU) in the reaction to the calibrator RLU, which is calculated by the system as an Index (S/C). The resulting chemiluminescent reaction is measured as a relative light unit (RLU) by the ARCHITECT i1000SR and i2000SR systems, or other authorized instruments. Results are interpreted as positive if an index (S/C) is ≥1.4 and as negative if <1.4.

### Neutralization assay

#### GenScript USA Inc., cPass SARS-CoV-2 Neutralization Antibody Detection Kit (sVNT)

The GenScript USA Inc., cPass SARS-CoV-2 Neutralization Antibody Detection Kit, also known as SARS-CoV-2 Surrogate Virus Neutralization Test (sVNT) kit, was performed according to the manufacturer’s instructions (GenScript USA Inc.,). Briefly, in separate tubes, a 1 : 1 ratio of sample or control to HRP conjugated to SARS-CoV-2 receptor binding domain fragment (HRP-RBD) were pre-incubated at 37 °C for 30 min. After adding the mixtures into capture plates pre-coated with human angiotensin converting enzyme 2 (hACE2), the plates were sealed with a plate sealer and incubated at 37 °C for 15 min. The plates were washed four times with wash solution, 100 µl of TMB Solution was added to each well, and the plates were incubated in the dark at 20–25 °C for 15 min. Following the addition of 50 µl of stop solution to each well, the absorbance was immediately read using a PerkinElmer Victor XV plate reader at 450 nm. This assay detects serum or plasma antibodies that neutralize the RBD-hACE2 interaction and are interpreted by the inhibition rate, calculated using the following formula: Inhibition (%) = (1 − sample optical density value/negative control optical density value) × 100. Specimens are considered positive for neutralizing antibodies against SARS-CoV-2 RBD if the percent inhibition is ≥20 % and negative (or not detectable) if the percent inhibition is <20 %.

### Microneutralization assay (MNT)

MNT tests were performed using a mNeonGreen reporter SARS-CoV-2, a gift from Pei-Yong Shi (University of Texas Medical Branch) [20]. Sera were gamma-irradiated with 2×10^6^ Rads, then heat-inactivated at 56°C for 1 h. The day prior to the assay, 3×10^4^ Vero (ATCC CCL-81) cells were seeded in each well of clear-bottom, black-walled 96-well plates in Fluorobrite DMEM (Thermo Fisher Scientific) supplemented with 10 % (v/v) heat-inactivated fetal calf serum (HI-FCS). The next day, seven point, three-fold serial dilutions of sera were generated in Fluorobrite DMEM supplemented with 2 % (v/v) HI-FCS. The mNeonGreen-SARS-CoV-2 virus stock was diluted in Fluorobrite DMEM +2 % HI-FCS, mixed with each sera dilution, incubated at 37 °C for 30 min, then added to the cells. Triplicate technical replicates were performed for each dilution of serum (from 1 : 10 to 1 : 7290), with the final multiplicity of infection being 0.1. Twenty-four hours post-infection, the media was removed from each well and replaced with DMEM +10 % (v/v) FCS. The mNeonGreen fluorescence in each well was read using a BioTek Cytation 3. Following normalization using virus-only and no-virus control wells, GraphPad Prism (RRID:SCR_002798) was used to fit a four-parameter equation to semi-log plots of the data and derive the dilution of sera that inhibited 50 % of the fluorescent signal (50 % inhibitory dilution; ID_50_).

### Statistical analysis

Statistical analysis was performed using GraphPad Prism (RRID:SCR_002798) seven software. Sensitivity and specificity were calculated using the rRT-PCR results as the reference standard. Pearson’s correlation analysis was used to assess the comparative analyses between SARS-CoV-2 immunoassays and the MNT assay, as well as the sVNT assay and the MNT assay.

## Results

The overall positivity rate results for all specimens across three immunoassays in comparison with the reference standards are summarized in Table 1. To assess the agreement between methods, each of the 204 specimens were tested using the SARS-CoV-2 in-house ELISA (S ectodomain), Ortho VITROS Immunodiagnostic Products Anti-SARS-CoV-2 Total Reagent Pack assay (S1 domain), and the Abbott SARS-CoV-2 IgG assay (N protein). The results of each immunoassay were compared to the reference standard, the rRT-PCR results, and the negative control group (Table 2). Most specimens showed complete agreement with the reference standard. Of the 204 specimens, five specimens (2.45 %) were discordant when using the in-house ELISA, three specimens (1.47 %) were discordant when using the Ortho assay, and five specimens (2.45 %) were discordant when using the Abbott assay. This resulted in similar accuracy rates among all three immunoassays (Table 2).

**Table 1.** Comparison of positivity rate detected by reference standard* and immunoassays

Rate (*n*=204)	Reference standard	ELISA spike ectodomain	Ortho	Abbott
Positive	87 (42.6 %)	86 (42.2 %)	84 (41.2 %)	84 (41.2 %)
Negative	117 (57.3 %)	118 (57.8 %)	120 (58.8 %)	120 (58.8 %)

*Patients rRT-PCR positive or negative for SARS-CoV-2.

**Table 2.** Agreement between reference standards and SARS-CoV-2 immunoassays

	ELISA spike ectodomain		Ortho		Abbott	
	**PCR+**	**PCR-**	**PCR+**	**PCR-**	**PCR+**	**PCR-**
T+	84	2	84	0	83	1
T-	3	115	3	117	4	116
Total	204		204		204	
	**ELISA spike ectodomain (95 % CI)**		**Ortho (95 % CI)**		**Abbott (95 % CI)**	
Sensitivity	96.55 (90.25–99.28)		96.55 (90.25–99.28)		95.4 (88.64–98.73)	
Specificity	83.76 (75.81–89.93)		99.15 (95.33–99.98)		99.15 (95.33–99.98)	
% Discordant	2.45 (5)		1.47 (3)		2.45 (5)	
Accuracy	97.6		98.6		97.6	

*T+ Immunoassay Test positive, T- Immunoassay Test negative.

**Fig. 1.**
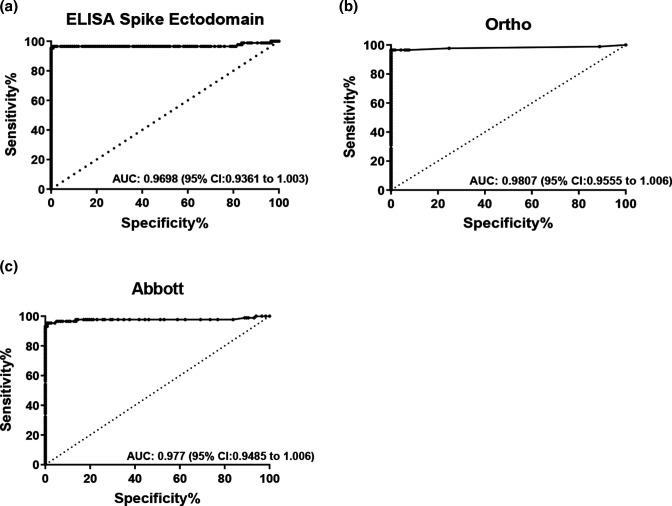
Receiver Operating Characteristic (ROC) Curves for each Immunoassay. (**a**) ELISA Spike Ectodomain Total Ab, (**b**) Ortho VITROS Immunodiagnostic Products Anti-SARS-CoV-2 Total Reagent Pack Ab, (**c**) Abbott SARS-CoV-2 IgG Ab. Dashed lines represent assay performance at specific cut off values. An AUC of 0.9–1.0 is considered excellent, 0.8–0.9 very good, 0.7–0.8 good, 0.6–0.7 sufficient, <0.5 test not useful [24].

Receiver-operating characteristic (ROC) area under the curve (AUC) analysis for the in-house ELISA, Ortho, and the Abbott assays were performed to assess assay performance characteristics. When using the established cutoff value determined by the manufacturer for each assay, all three assays showed a sensitivity above 95% (Fig. 1a, c). The in-house ELISA and the Ortho assay had the highest sensitivity of 96.55 % (95 % CI 90.25–99.28 %), see Table 2. The Abbott assay had the lowest sensitivity with 95.4 % (95 % CI 88.64–98.73 %). Specificity was highest with the Ortho and Abbott assay being 99.15 % (95 % CI 95.33–99.98), and lowest with the in-house ELISA being 83.76 % (95 % CI 75.81–89.93), see Table 2. The area under the curve was high for all three assays with the highest AUC being Ortho (0.9807) followed by Abbott (0.977) and the in-house ELISA (0.9698) (Fig. 1).

**Fig. 2.**
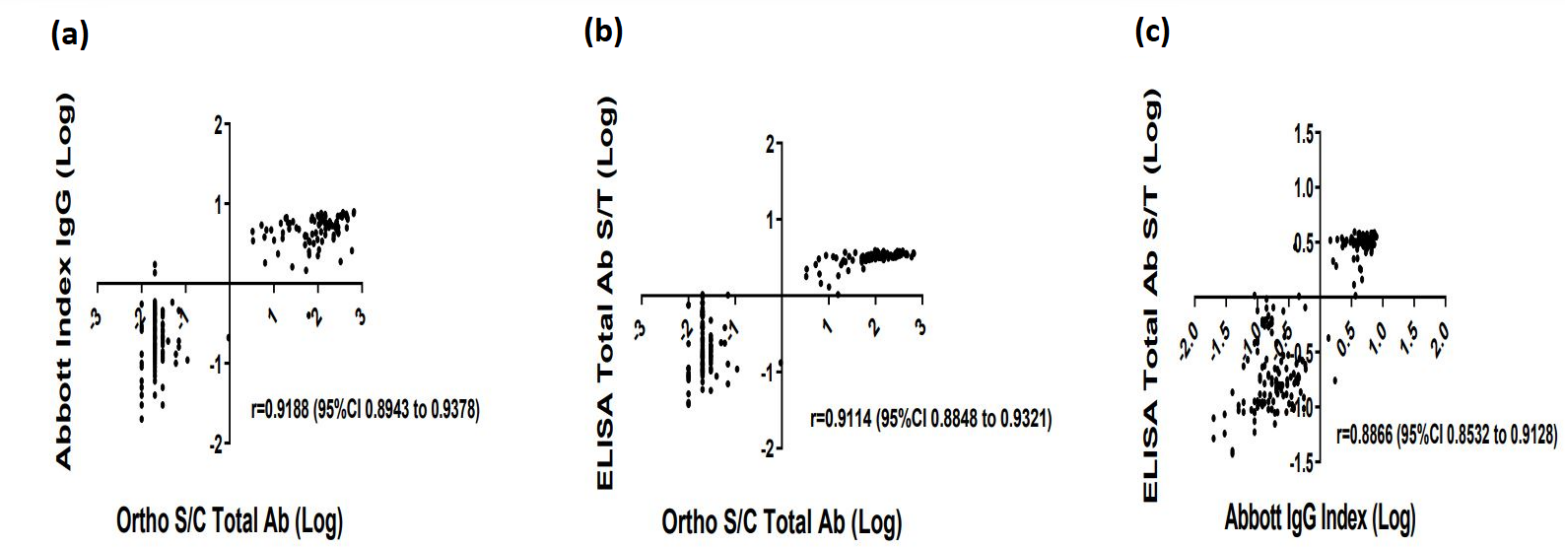
Correlational analysis of the numerical values obtained by each Immunoassay. (**a**) Abbott Index IgG values vs Ortho S/C Total Ab values, (**b**) ELISA Spike Ectodomain Total Ab S/T values vs Ortho S/C Total Ab values, (**c**) ELISA Spike Ectodomain Total Ab S/T values vs Abbott Index IgG values. Pearson correlation coefficient (**r**), and 95 % Confidence Interval (CI) are indicated for each immunoassay plotted against each other. Data are presented for 87 rRT-PCR confirmed SARS-CoV-2 specimens known as the positive reference standard and 117 negative specimens known as the negative reference standard. Signal to Calibrator, (S/C); Signal to Threshold (S/T).

**Fig. 3.**
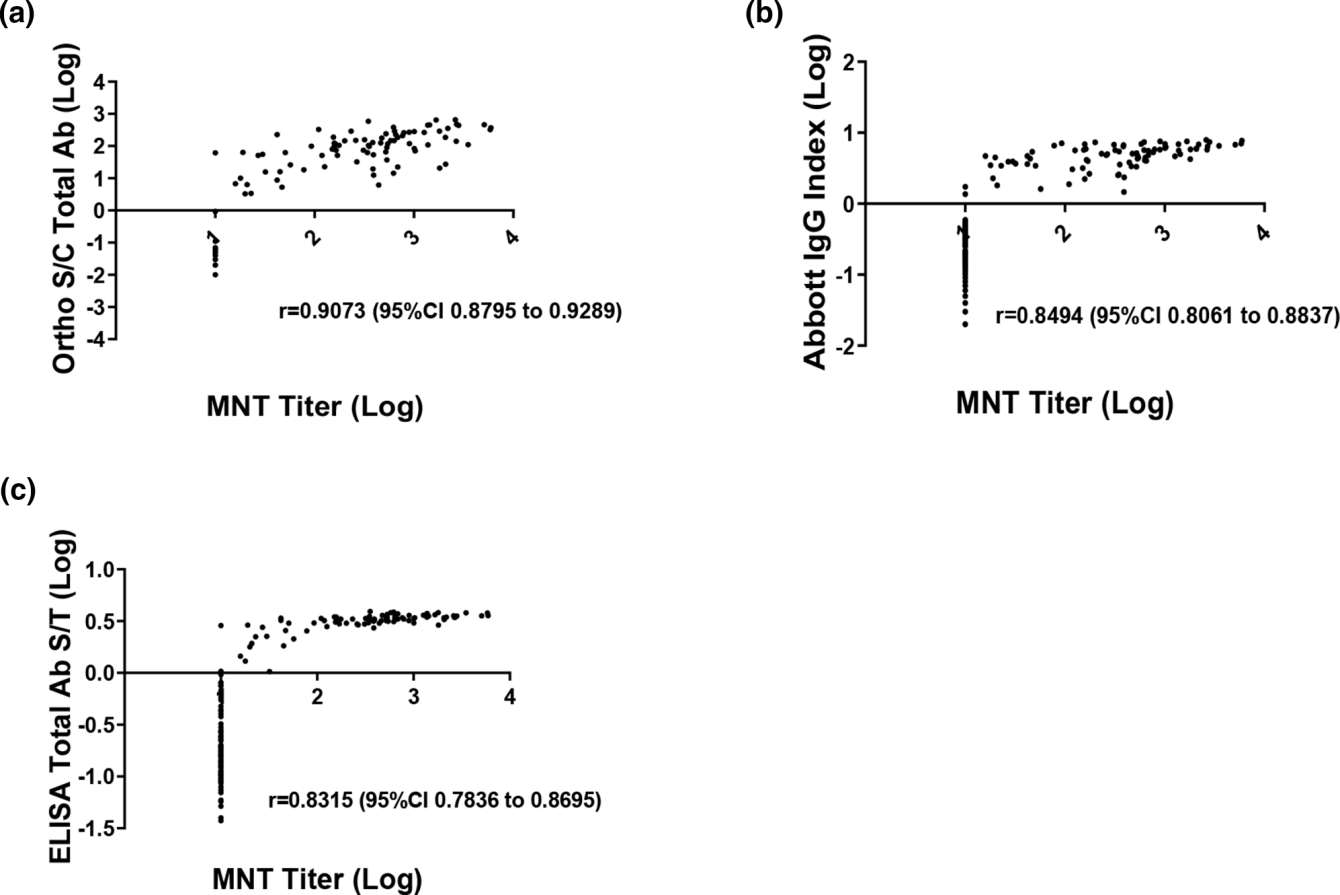
Correlational analysis between microneutralization test (MNT) titers and Immunoassays numerical values. (**a**) Ortho S/C Total Ab values vs. MNT titer, (**b**) Abbott Index IgG values vs MNT titer, (**c**) ELISA Spike Ectodomain Total Ab S/T values vs MNT titer. Pearson correlation coefficient (**r**), and 95 % Confidence Interval (CI) are indicated for each immunoassay values plotted against the MNT titer. Data are presented for 87 rRT-PCR confirmed SARS-CoV-2 specimens known as the positive reference standard and 117 negative specimens known as the negative reference standard.

As shown in Fig. 2, correlation of the quantitative values between each assay were assessed by Pearson correlation coefficient statistical analysis. The quantitative values of each assay correlated highly with one another. There was a strong, significant positive correlation found between Ortho S/C values and Abbott Index values with a r value of 0.9188 (95 % CI 0.8943–0.9378) (Fig. 2a). The positive correlation between Ortho S/C values and in-house ELISA S/T values was also significant with an r value of 0.9114 (95 % CI 0.8848–0.9321) (Fig. 2b). The Abbott Index values showed a slightly less significant correlation with the in-house ELISA S/T values with an r value of 0.8866 (95 % CI 0.8532–0.9128) (Fig. 2c). Patient specimens were also tested to evaluate the correlation between titers generated by the microneutralization test and quantitative values generated by the immunoassays. A significant correlation was found between Ortho S/C values and MNT titers with a r value of 0.9073 (95 % CI 0.8795–0.9289) (Fig. 3a). Both the Abbott and in-house ELISA assay values showed a lower degree of positive correlation with the MNT test titers with an r value of 0.8494 (95 % CI 0.8061–0.8837) and 0.8315 (95 % CI 0.7836–0.8695), respectively (Fig. 3b, c).

**Fig. 4.**
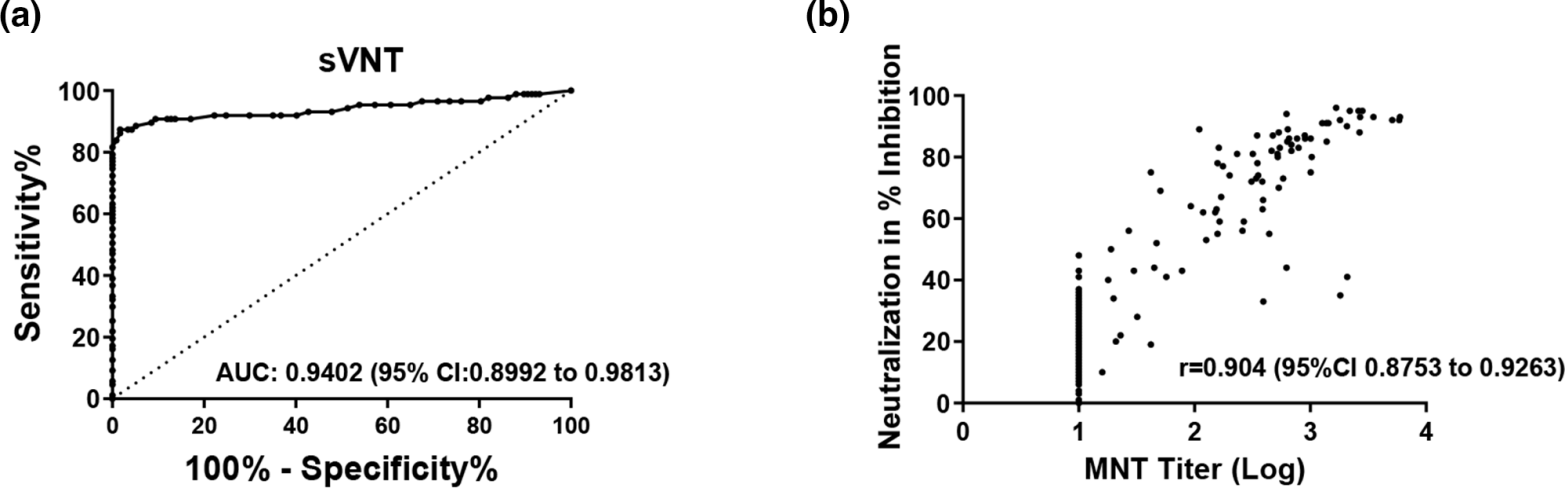
Surrogate Virus Neutralization Test (sVNT) performance analysis. (**a**) ROC curve for sVNT, (**b**) Correlational Analysis between sVNT percent inhibition and MNT titer. Pearson correlation coefficient (**r**), and 95 % Confidence Interval (CI) are indicated for each sVNT percent inhibition values plotted against the MNT titer. Data are presented for 87 rRT-PCR confirmed SARS-CoV-2 specimens known as the positive reference standard and 117 negative specimens known as the negative reference standard. Dashed lines represent assay performance at specific cut off values. An AUC of 0.9–1.0 is measured as being excellent, 0.8–0.9 very good, 0.7–0.8 good, 0.6–0.7 sufficient, <0.5 test not useful [24].

To investigate the neutralizing antibody capacity of each sample without the use of viable virus and a high containment facility, the performance characteristics of the SARS-CoV-2 Surrogate Virus Neutralization Test (sVNT) [21], which measures the antibody-mediated inhibition of SARS-CoV-2 RBD-ACE2 interaction, was examined. ROC curve analysis indicated significant performance with an AUC of 0.9402 (Fig. 4a). At the manufacturer established cut off (inhibition >20 %), the sensitivity is 94.25 % (95 % CI 87.1–98.11 %), while the specificity is 48.72 % (95 % CI 39.37–58.13 %) (Table 3). If the sVNT cut-off was adjusted to 40%, the specificity of the assay would increase to 98.29 % (95 % CI 93.96–99.79 %) and reduce the sensitivity to 86.21 % (95 % CI 77.15–92.66 %). Next, the MNT test titers and sVNT inhibition percentage rate results were plotted against each other and showed a high degree of positive correlation with an r value of 0.904 (95 % CI 0.8753–0.9263) (Fig. 4b). The inhibition rate by sVNT was directly related to the MNT titers, on average increasing from 19 % for specimens with a titer of 1 : 10 to 70 % for specimens with a titer greater than 1 : 10 (data not shown). Using the MNT test as the reference standard for neutralizing antibodies, 64 specimens (31.4 %) were discordant when using the sVNT test at the manufacturer’s cutoff. However, using the adjusted cutoff of 40 %, reduced the discordance to 5.9 % (Table 4).

**Table 3.** Sensitivity and specificity of Surrogate Virus Neutralization Test (sVNT)

	sVNT (20%)	sVNT (40%)
Sensitivity	94.25 (87.1–98.11 %)	86.21 (90.25–99.28)
Specificity	48.72 (39.37–58.13 %)	98.29 (95.33–99.98)

**Table 4.** Comparison of positivity rate detected by neutralization assays

	sVNT (20%)		sVNT (40%)		Discordance	
	**MNT(+)**	**MNT(-)**	**MNT(+)**	**MNT(-)**	**% Discordant (n)**	
T+	79	63	73	5	31.4 (64)	20 %
T-	1	61	7	119	5.9 (12)	40 %
Total	204		204			
Rate (*n*=204)	**MNT**		**sVNT (20%)**		**sVNT (40%)**	
(+) Reference Standard*	80 (39.2 %)		142 (69.6 %)		78 (38.2 %)	
(-) Reference Standard*	12 4(60.8 %)		62 (30.4 %)		126 (61.8 %)	

*Patients rRT-PCR positive (+) or negative (-) for SARS-CoV-2.

MNT, Microneutralization Assay; sVNT, Surrogate Virus Neutralization Test; T, Test.

## Discussion

Serological tests serve as valuable tools in the detection of SARS-CoV-2 antibodies. The ongoing COVID-19 pandemic has increased the need for the development and evaluation of accurate tools that can aid in SARS-CoV-2 serosurveillance and therapeutic studies. The presence of SARS-CoV-2 specific antibodies can be an indication of a present or past infection or vaccination. Furthermore, to understand correlates of immunity and protection, the functionality of the elicited antibody responses to SARS-CoV-2 is important. Within a short period of time, a multitude of serology platforms for SARS-CoV-2 antibody detection have become available through EUA from the FDA [18]. In addition, an immunoassay detecting virus‐specific neutralizing antibodies became available [21]. Although serological tests are not recommended for clinical diagnosis of SARS-CoV-2, given the importance of interpreting results from serological tests, independent evaluation of the performance characteristics of these serological tests can be crucial in providing reliable information that may help support surveillance, accurate diagnosis, treatment, and prevention of SARS-CoV-2 infections.

In the present study, we used a panel of serum specimens from 87 rRT-PCR confirmed SARS-CoV-2 positive patients, 18 rRT-PCR SARS-CoV-2 negative patients and 99 pre-COVID-19 negative controls to evaluate the performance of an in-house ELISA (S ectodomain), as well as three commercially available assays, namely the Ortho VITROS Immunodiagnostic Products Anti-SARS-CoV-2 Total Reagent Pack assay (S1 domain), the Abbott SARS-CoV-2 IgG assay (N protein), and the GenScript Surrogate Virus Neutralization Test (RBD). The study described here evaluates the performance between the in-house pre-fusion stabilized ectodomain of SARS-CoV-2 S ELISA and commercially available Ortho VITROS Immunodiagnostic Products Anti-SARS-CoV-2 Total Reagent Pack and Abbott SARS-CoV-2 IgG CLIA assays. Despite using different SARS-CoV-2 antigens, our results revealed that in comparison to the rRT-PCR results there were no significant differences between the assays ability to detect SARS-CoV-2 specific antibodies; as indicated by the percent positivity rates, comparable accuracy rates, and overlapping confidence intervals (Tables 1 and 2). Among the three immunoassays, using rRT-PCR as the reference standard, the Orthro assay had the highest sensitivity (96.55 %) and specificity (96.55 %) values. Although the in-house ELISA had high sensitivity (96.55 %), it had the lowest specificity (83.76 %) with two false positives and one false negative. Despite having a high specificity (99.15 %) and sensitivity (95.4 %) the Abbott assay had one false positive and four false negatives. With similar sensitivity and specificity to the Abbott assay, the Ortho assay had no false positives and three false negatives. This could be due to antibody responses being below the assays detection threshold at the time of testing, or an initial false positive rRT-PCR result. In line with our study, results reported by a large study evaluating performance characteristics of five immunoassays at various disease prevalences demonstrated lower numbers of false positive tests per million and higher numbers of false-negative tests per million with high specificity platforms such as the Abbott assay when compared to other assays [22]. Testing for SARS-CoV-2 specific antibodies with highly specific serological tests, such as the Abbott or Orthro assay, warrants a two-tier testing strategy incorporating a screening and confirmatory testing method when interpreting negative results from patients who are symptomatic.

Receiver-operating characteristic (ROC) area under the curve (AUC) analysis is a measure of diagnostic accuracy and helps estimate how well the serological test differentiates those with and without the disease in question [23]. ROC AUC analysis (Fig. 1) indicated that all three immunoassays utilized in this study performed well in differentiating between the presence and absence of SARS-CoV-2 specific antibodies in serum. Although, AUC analysis does not consider disease prevalence, it provides a meaningful interpretation of the general assessment for comparing two or more serological tests. In this study, statistical analysis of the quantitative values of the immunoassays showed a high degree of correlation amongst all immunoassays (Fig. 2). More importantly, a positive test result for SARS-CoV-2 specific Total or IgG antibody obtained with the immunoassays positively correlated with the presence of neutralizing antibodies, thus demonstrating the usefulness of evaluating multiple immunogenic antigens. Overall, our results showed that there was a moderate to high correlation between the quantitative values of the immunoassays and the MNT titers (Fig. 3). The microneutralization assay is a highly sensitive and specific functional assay which serves as the gold standard for detecting SARS-CoV-2 specific neutralizing antibodies in sera. However, the MNT can only be performed in a BSL-3 facility and can be laborious and time consuming. Therefore, we performed an independent evaluation on the Surrogate Virus Neutralization Test (sVNT), to examine its performance as an alternative assay for measuring SARS-CoV-2 neutralizing antibodies. Our results revealed when using the rRT-PCR results as the reference standard and GenScript USA Inc. recommended cut off, sVNT had a high sensitivity of 94.25 (87.1–98.11 %) and a low specificity of 48.72 (39.37–58.13 %) (Table 3). A 31.4 % discordance was observed when analyzing the agreement between the MNT test results and the sVNT test at the manufacturer’s cutoff (Table 4). This variation could be explained in that the sVNT method only measures functional antibodies that have the capacity to neutralize SARS-CoV-2 RBD. Adjustments to the recommended cut off value increased the specificity and decreased the sensitivity of the sVNT test. This adjustment also decreased discordance between the two neutralization assays to 5.9 % (Table 4). Increasing the recommended cutoff values to improve specificity at the expense of sensitivity when assessing serological tests performances has been reported [24].

Our study is limited by the lack of detailed clinical information available; samples are deidentified and all information may not be available regarding age, gender, the date from symptom onset in COVID-19 patients and rRT-PCR testing. Therefore, the data could not be correlated with symptoms or disease severity. SARS-CoV-2 serological tests cannot determine active infection and are limited by their ability to only detect whether a person has produced antibodies in response to SARS-CoV-2 virus, thus timing of sample collection and testing after disease onset is important. Some studies have reported an increase in effectiveness of serological tests to detect antibodies against SARS-CoV-2 after the first week of symptom onset [25–27]. Bryan *et al.*, examined the sensitivity of the Abbott assay from date of symptom onset and found that the sensitivity of the assay was 53.1 % at 7 days, 82.4 % at 10 days, 96.9 % at 14 days and 100 % at 17 days after symptom onset [25]. This study further demonstrated that the sensitivity of the Abbott assay from the date of rRT-PCR positivity was 88.7 % at 7 days, 97.2 % at 10 days, 100 % at 14 days, and 100 % at 17 days post-symptom onset [25]. It is possible that the variation between the assays ability to detect SARS-CoV-2 antibodies is due to specimens being collected at a relatively early stage of infection. In some individuals, seroconversion is delayed or does not occur at all, thus contributing to an assay ability to produce false-negative results [28–30]. Furthermore, the discordance between the assays may be associated with the ability of the test to detect the presence of antibodies against the spike (S) protein as opposed to antibodies against the nucleocapsid (N) protein [31].

In conclusion, the current results demonstrate that the three immunoassays compared in this study are practical and well suited for detecting SARS-CoV-2 binding antibodies. In addition, the immunoassays are convenient methods with short turnaround times. However, turnaround times may vary from one or 2 h to one or 2 days, depending on the workload. Furthermore, this study indicates that GenScript USA Inc., cPass SARS-CoV-2 Neutralization Antibody Detection Kit also known as SARS-CoV-2 Surrogate Virus Neutralization Test (sVNT) kit is a useful method that could be easily performed to evaluate neutralizing antibodies against SARS-CoV-2. However, due to the assay’s limitations, more studies should be performed on an independent laboratory basis to define its performance characteristics and value. Further prospective studies utilizing a larger sample set and a standardized reference test should be done to independently evaluate the performance of serological tests.
